# Relationship between family–school–peer risks and problematic Internet use among boarding high school students in China: based on a latent profile analysis

**DOI:** 10.3389/fpsyt.2026.1874111

**Published:** 2026-06-08

**Authors:** Jing Shi, Bopeng Yu, Sihan Hao

**Affiliations:** 1 School of Educational Science, Shenyang Normal University, Shenyang, China; 2 Yingkou Vocational and Technical College, Yingkou, China

**Keywords:** boarding high school students, family-school-peer risks, latent profile analysis, maladaptive cognition, problematic internet use, psychological resilience

## Abstract

This study examined how configurations of family, school, and peer risks were associated with problematic Internet use among Chinese boarding high school students. A total of 736 students (54.21% male; *M*_age_ = 16.78, SD = 0.99) from two public boarding high schools in Yingkou, Liaoning Province, China, were recruited through cluster convenience sampling during the spring semester of 2025. Latent profile analysis was used to identify patterns of family–school–peer risks, and subsequent analyses examined differences in problematic Internet use across profiles, the mediating role of maladaptive cognition, and the moderating role of psychological resilience. Four profiles were identified: the Low Combined Risk Group (43.89%), the Balanced Risk Group (20.11%), the High School–Peer Risk Group (17.93%), and the High Combined Risk Group (18.07%). Problematic Internet use differed significantly across these profiles, with the High Combined Risk Group reporting the highest level. Maladaptive cognition partially mediated the association between high-risk profiles and problematic Internet use, and psychological resilience attenuated the direct association between risk profiles and problematic Internet use. These findings provide a person-centered perspective on family–school–peer risk configurations among boarding high school students and highlight the psychological processes associated with problematic Internet use.

## 1 Introduction

Problematic Internet use refers to an inability to control impulses to use the Internet and is associated with psychological, social, educational, and occupational problems (1, 2). Nowadays, digital technologies are intricately integrated into the daily lives and schoolwork of adolescents. The high school period with its significant cognitive development and self-identity formation yet limited self-regulation is a phase of heightened vulnerability to problematic Internet use (3).

Boarding high school students are physically separated from their families during much of the school term and simultaneously face academic competition, adaptation to communal living, and complex peer relationships (4, 5). These conditions may increase students’ reliance on the Internet for emotional regulation, recreation, and social contact (6). Therefore, focusing on boarding high school students is important for understanding problematic Internet use in a developmental context where family, school, and peer influences may be reorganized.

The boarding context may reshape adolescents’ exposure to family, school, and peer risks in ways that differ from those of non-boarding students. Because boarding students spend much of their daily life on campus, direct parental monitoring and immediate family support are relatively reduced, whereas school climate, teacher–student relationships, and peer interactions become more continuous and salient. Shared living arrangements may also intensify peer influence, including both deviant peer affiliation and peer victimization. In this context, family risks may operate together with school and peer risks, while school- and peer-related stressors may become especially central to students’ psychological adjustment. Boarding high school students therefore provide a meaningful population for examining how family, school, and peer risks are configured and how these configurations are associated with problematic Internet use.

According to Ecological Systems Theory, the family, school, and peer environments constitute the core microsystems that shape adolescents’ psychological and behavioral development (7). Prior research has shown that problematic Internet use is associated with multiple contextual risks, including low family socioeconomic status, parent–child conflict, teacher–student conflict, deviant peer affiliation, and peer victimization (8–10). However, previous studies have mainly concentrated on single risk factors within specific systems, generally neglecting the ways in which multi-system risks may be combined within individuals. The Demands–Resources Model suggests that environmental demands rarely operate in isolation; rather, they may converge and jointly relate to individual adjustment (11). Therefore, latent profile analysis (LPA) is useful for identifying underlying patterns of family–school–peer risks and for examining how these patterns are associated with problematic Internet use.

Moreover, the Cognitive–Behavioral Model helps explain the psychological processes linking environmental risks to problematic Internet use. Distal environmental risks in family, school, and peer systems may be associated with problematic Internet use through maladaptive cognition as a proximal psychological factor (12). Maladaptive cognition generally involves self-doubt, negative self-evaluation, and pessimistic perceptions of reality. Such cognitive patterns may be more likely to emerge in the context of familial dysfunction, school challenges, and peer conflict (13, 14). Once formed, maladaptive cognitive patterns may strengthen adolescents’ reliance on the Internet as a means of compensation or escape (15, 16). This framework suggests that different risk configurations may be associated with problematic Internet use partly through maladaptive cognitive processes.

At the same time, psychological resilience represents an important individual protective factor. Resilience refers to the capacity to adapt to adversity and to maintain psychological functioning under stressful conditions (17–19). Previous studies have shown that adolescents with higher psychological resilience are generally better able to regulate negative emotional and cognitive responses, which may weaken the association between environmental risks and problematic Internet use (20, 21). Thus, psychological resilience may attenuate the direct association between family–school–peer risk profiles and problematic Internet use.

**Figure 1:**
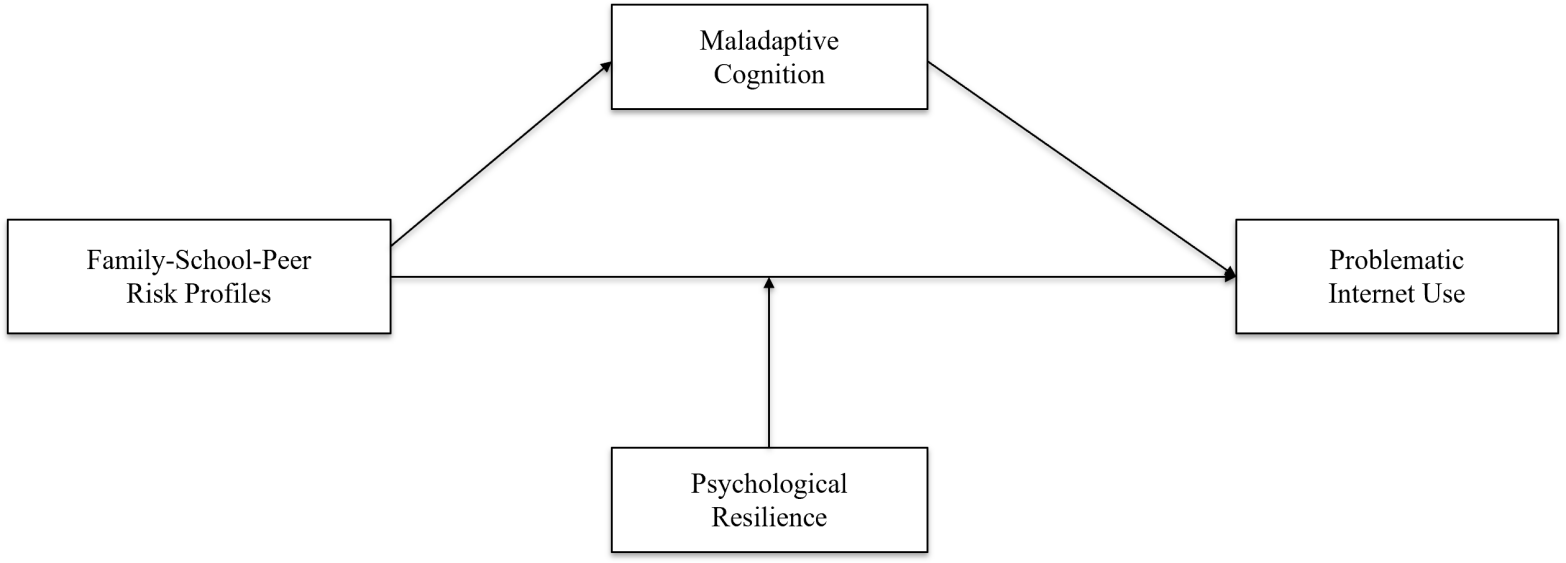
Theoretical framework.

Taken together, these perspectives provide a sequential framework for the present study. Ecological Systems Theory identifies the family, school, and peer systems as proximal developmental contexts for adolescents. The Demands–Resources Model further suggests that risks from these systems may not operate independently but may cluster into distinct configurations. The Cognitive–Behavioral Model explains why these risk configurations may be associated with problematic Internet use through maladaptive cognition. Finally, resilience theory suggests that psychological resilience may function as an individual protective factor that weakens the association between environmental risk profiles and problematic Internet use. The theoretical framework of the present study is presented in **Figure 1**.

In summary, the current study integrates Ecological Systems Theory, the Demands–Resources Model, and the Cognitive–Behavioral Model to examine problematic Internet use among boarding high school students. This study first employed LPA to identify latent patterns of family–school–peer risks, then examined differences in problematic Internet use across profiles, and finally tested maladaptive cognition as a mediator and psychological resilience as a moderator of the direct association between risk profiles and problematic Internet use. The findings are expected to provide a person-centered understanding of environmental risk heterogeneity and individual psychological differences among boarding high school students.

## 2 Method

### 2.1 Participants and procedure

Participants were recruited from two public boarding high schools in Yingkou, Liaoning Province, China, during the spring semester of 2025. Cluster convenience sampling was used. Before data collection, permission was obtained from the participating schools. Because the sample included both minors and adult students, consent procedures were implemented according to participants’ age. For students younger than 18 years, oral consent or assent was first obtained during recruitment and written informed consent from parents or legal guardians and written assent from students were obtained before the formal survey. For students aged 18 years or older, oral agreement was first obtained, and written informed consent was obtained directly from the students before participation. Students were informed that participation was voluntary, that their responses would remain anonymous and confidential, and that they could withdraw at any time.

The questionnaire was administered offline using paper-and-pencil questionnaires. Trained researchers distributed and collected the questionnaires in classroom settings following standardized instructions. The questionnaire took approximately 10 to 15 min to complete. Before completing the questionnaire, students received an explanation of the study purpose, confidentiality procedures, and response requirements. A total of 798 questionnaires were collected. During screening, 62 questionnaires were excluded, leaving 736 valid questionnaires for analysis (effective response rate = 92.23%). Among the excluded questionnaires, 38 were excluded because they contained excessive missing responses and 24 were excluded because they showed patterned responses. Excessive missing responses were defined as any unanswered item on the questionnaire; therefore, questionnaires with one or more missing item responses were excluded. Patterned responses were defined as evidently non-substantive response patterns, including repeated identical responses across nearly all items or other regular response patterns inconsistent with item content. No missing values remained in the final analytic dataset. Using available demographic information in the original dataset, excluded and retained questionnaires did not differ significantly in gender, age, grade, or family structure, *p*s > 0.05.

The final sample consisted of 736 students, namely, 399 male (54.21%) and 337 female students (45.79%). Participants ranged in age from 15 to 19 years, with a mean age of 16.78 years (SD = 0.99). The sample included 242 Grade 10 students (32.88%), 253 Grade 11 students (34.38%), and 241 Grade 12 students (32.74%). Gender, age, and grade were included as covariates in subsequent analyses where appropriate.

### 2.2 Measures

The measures used in the present study were established instruments or measures previously developed, validated, or used in Chinese adolescent samples. For each measure, the source, scoring procedure, and internal consistency coefficient in the current sample are reported below.

#### 2.2.1 Family–school–peer risks

Risk indicators were selected to represent major environmental risks in the family, school, and peer microsystems of boarding adolescents. Within each system, we included two indicators that captured different forms of risk. In the family system, low family socioeconomic status represented a structural background risk, whereas parent–child conflict represented a relational family risk. In the school system, low school connectedness represented weak school belonging, whereas teacher–student conflict represented interpersonal tension in the school context. In the peer system, deviant peer affiliation represented exposure to risky peer norms, whereas peer victimization represented negative peer experiences. These six indicators were chosen because they are theoretically relevant to adolescent adjustment, developmentally meaningful for boarding high school students, and sufficiently distinct to describe heterogeneous family–school–peer risk configurations through LPA (5, 7, 8, 10, 22–29).

##### 2.2.1.1 Low family socioeconomic status

Low family socioeconomic status was treated as a composite family background risk indicator rather than a conventional latent psychological scale. It was assessed using four five-point items: father’s education level, mother’s education level, family economic condition, and family structure. Item scores were coded so that higher scores indicated greater socioeconomic disadvantages. The mean of the four items was used as the low family socioeconomic status indicator.

##### 2.2.1.2 Parent–child conflict

Parent–child conflict was evaluated using the Parent–Child Conflict Questionnaire (23), comprising eight items rated on a five-point Likert scale (1 = never to 5 = always). Higher total scores represented more frequent parent–child conflicts. Cronbach’s α was 0.86 in this study.

##### 2.2.1.3 Low school connectedness

Low school connectedness was assessed using the School Connectedness Scale (24), comprising five items rated on a five-point Likert scale (1 = strongly disagree to 5 = strongly agree). Related research has also used school connectedness to examine adjustment among Chinese adolescents (25). After reverse scoring, higher total scores indicated lower levels of school connectedness. Cronbach’s α was 0.88 in this study.

##### 2.2.1.4 Teacher–student conflict

Teacher–student conflict was evaluated using the conflict subscale of the Teacher–Student Relationship Scale (26), comprising six items rated on a five-point Likert scale (1 = strongly disagree to 5 = strongly agree). Higher total scores indicated more severe teacher–student conflicts. Cronbach’s α was 0.96 in this study.

##### 2.2.1.5 Deviant peers

Deviant peer affiliation was evaluated using the Deviant Peer Questionnaire (27), comprising eight items rated on a five-point Likert scale (1 = none to 5 = all). Higher total scores indicated closer affiliations with deviant peers. Cronbach’s α was 0.93 in this study.

##### 2.2.1.6 Peer victimization

Peer victimization was assessed via the Peer Victimization Questionnaire (28), comprising nine items rated on a five-point Likert scale (1 = never to 5 = ≥4 times). Higher total scores represented more severe peer victimization experiences. Peer victimization has also been examined in Chinese adolescent research on problematic online game use (29). Cronbach’s α was 0.98 in this study.

#### 2.2.2 Maladaptive cognition

Maladaptive cognition was evaluated using the Adolescent Maladaptive Cognition Scale (30), comprising 12 items rated on a five-point Likert scale (1 = strongly disagree to 5 = strongly agree). Higher total scores indicated elevated levels of maladaptive cognition. Cronbach’s α was 0.77 in this study.

#### 2.2.3 Problematic Internet use

Problematic Internet use was evaluated using the Adolescent Pathological Internet Use Scale (31), comprising 38 items rated on a five-point Likert scale (1 = strongly disagree to 5 = strongly agree). Higher total scores indicated more severe problematic Internet use. Cronbach’s α was 0.94 in this study.

#### 2.2.4 Psychological resilience

Psychological resilience was evaluated using the Psychological Resilience Scale (32), comprising 27 items rated on a five-point Likert scale (1 = strongly disagree to 5 = strongly agree). Higher total scores indicated stronger psychological resilience. Cronbach’s α was 0.95 in this study.

### 2.3 Statistical analysis

Data analyses were conducted using SPSS 26.0, Mplus 8.3, and PROCESS macro version 4.1. Descriptive statistics, reliability analyses, common method bias testing, analysis of variance (ANOVA), and *post-hoc* comparisons were performed in SPSS 26.0. *Post-hoc* comparisons were conducted using Fisher’s least significant difference (LSD) procedure.

LPA was conducted in Mplus 8.3 using the six standardized family–school–peer risk indicators: low family socioeconomic status, parent–child conflict, low school connectedness, teacher–student conflict, deviant peer affiliation, and peer victimization. Models with one to five profiles were estimated using robust maximum likelihood estimation (MLR) and the EMA optimization algorithm. The model used 20 initial-stage random starts and four final-stage optimizations. Class-specific means were freely estimated, within-class variances were constrained to be equal across classes, and within-class covariances were fixed to zero. The retained four-profile model terminated normally, and the best log-likelihood value was replicated across final-stage optimizations. Model selection was based on multiple criteria, including AIC, BIC, aBIC, Entropy, LMR, BLRT, class size, interpretability, and theoretical coherence. Lower AIC, BIC, and aBIC values indicated better model fit, whereas Entropy values closer to 1 indicated better classification accuracy. LMR and BLRT were used to compare models with *k* and *k*−1 profiles. The final solution was selected by considering statistical fit, class size, classification stability, and substantive interpretability.

After the latent profiles were identified, profile membership was dummy-coded, with the Low Combined Risk Group as the reference group. Relative mediation analyses were conducted using PROCESS Model 4 to examine whether maladaptive cognition mediated the association between profile membership and problematic Internet use. Psychological resilience was tested using PROCESS Model 5 as a moderator of the direct association between profile membership and problematic Internet use. All continuous variables were standardized before PROCESS analyses, and the reported regression coefficients are standardized coefficients. Indirect effects were evaluated using 5,000 bootstrap samples with 95% percentile bootstrap confidence intervals. Gender, age, and grade were included as covariates. Therefore, the model was described as a relative mediation model with moderation of the direct association rather than as a full moderated mediation model.

## 3 Results

### 3.1 Common method bias

Because all main variables were collected through self-report questionnaires at a single time point, common method bias was examined using Harman’s single-factor test. The results showed that the first unrotated factor accounted for 24.44% of the total variance, which was below the 40% reference value. This suggests that common method bias was unlikely to seriously distort the present findings.

### 3.2 Descriptive statistics

Overall descriptive statistics were first examined for the main study variables. The mean scores for the six family–school–peer risk indicators were as follows: low family socioeconomic status, *M* = 2.81, SD = 0.75; parent–child conflict, *M* = 2.49, SD = 1.03; low school connectedness, *M* = 2.68, SD = 1.37; teacher–student conflict, *M* = 2.79, SD = 1.51; deviant peer affiliation, *M* = 2.00, SD = 0.93; and peer victimization, *M* = 2.73, SD = 1.66. For the key psychological and behavioral variables, the mean scores were *M* = 2.38, SD = 0.78 for maladaptive cognition, *M* = 2.98, SD = 1.03 for psychological resilience, and *M* = 2.29, SD = 0.71 for problematic Internet use.

### 3.3 Joint latent profile analysis of family–school–peer risks

Joint LPA was employed to identify combination patterns among six risk factors across the family (low family socioeconomic status and parent–child conflict), school (low school connectedness and teacher–student conflict), and peer (deviant peers and peer victimization) systems. Models with one to five profiles were estimated. As shown in **Table 1**, AIC, BIC, and aBIC decreased as the number of profiles increased from one to five. The five-profile solution showed the lowest information criteria; however, it produced a very small profile accounting for only 3.26% of the sample, which raised concerns about class stability and substantive interpretability. In contrast, the four-profile solution showed adequate class sizes, clear substantive meaning, and high classification quality. The entropy value of the four-profile solution was 0.988, and the average posterior probabilities for the most likely class membership were 0.993, 0.999, 1.000, and 0.986, suggesting clear separation among the profiles. Therefore, considering statistical fit, class size, classification quality, and theoretical interpretability, the four-profile solution was retained as the final model.

**Table 1:** Fit indices for latent profile analysis of environmental risks.

Model	AIC	BIC	aBIC	Entropy	LMR(*p*)	BLRT(*p*)	Latent class proportions
1	12,552.70	12,607.92	12,569.82	—	—	—	—
2	9,552.99	9,640.42	9,580.08	0.984	<0.001	<0.001	0.62/0.38
3	8,129.59	8,249.23	8,166.67	0.987	<0.001	<0.001	0.59/0.23/0.18
4	6,935.10	7,086.94	6,982.16	0.988	0.001	<0.001	0.44/0.20/0.18/0.18
5	6,420.56	6,604.61	6,477.60	0.994	0.008	<0.001	0.17/0.43/0.03/0.18/0.18

**Figure 2:**
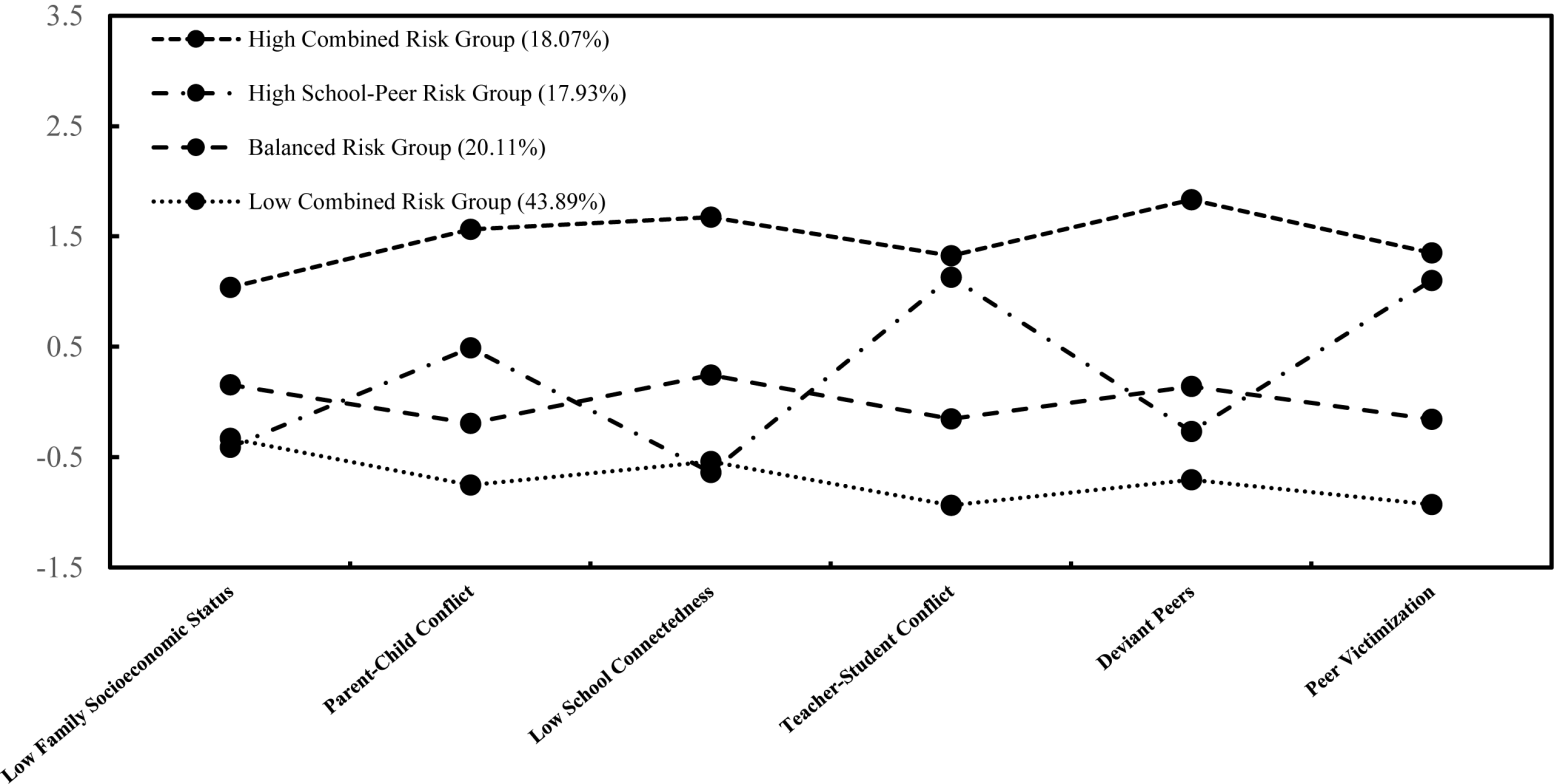
Joint latent profile plot of environmental risks. Values represent standardized scores of the six profile indicators. Higher scores indicate higher levels of risk.

As shown in **Figure 2**, the risk characteristics of each group were as follows: Class 1 showed universally low risk levels and was named the Low Combined Risk Group (43.89%). Class 2 showed moderate risk levels across systems and was named the Balanced Risk Group (20.11%). Class 3 had prominent risks primarily within the school and peer domains and was named the High School–Peer Risk Group (17.93%). Class 4 showed high risk levels across all three systems and was named the High Combined Risk Group (18.07%). **Figure 2** presents standardized scores of the six profile indicators. Beyond the profile-defining risk indicators displayed in **Figure 2**, descriptive statistics for the key psychological and behavioral variables across the four profiles further showed clear group differences. Problematic Internet use was lowest in the Low Combined Risk Group (*M* = 2.02, SD = 0.65), followed by the Balanced Risk Group (*M* = 2.35, SD = 0.68), the High School–Peer Risk Group (*M* = 2.47, SD = 0.56), and the High Combined Risk Group (*M* = 2.69, SD = 0.73). A similar pattern was observed for maladaptive cognition, with the lowest level in the Low Combined Risk Group (*M* = 2.15, SD = 0.72), followed by the Balanced Risk Group (*M* = 2.29, SD = 0.79), the High School–Peer Risk Group (*M* = 2.56, SD = 0.84), and the High Combined Risk Group (*M* = 2.89, SD = 0.54). Psychological resilience showed the opposite pattern: it was highest in the Low Combined Risk Group (*M* = 3.51, SD = 0.80) and lower in the Balanced Risk Group (*M* = 2.38, SD = 0.98), the High School–Peer Risk Group (*M* = 2.66, SD = 1.04), and the High Combined Risk Group (*M* = 2.67, SD = 0.96).

### 3.4 Group differences in problematic Internet use: ANOVA and *post-hoc* comparisons

**Figure 3:**
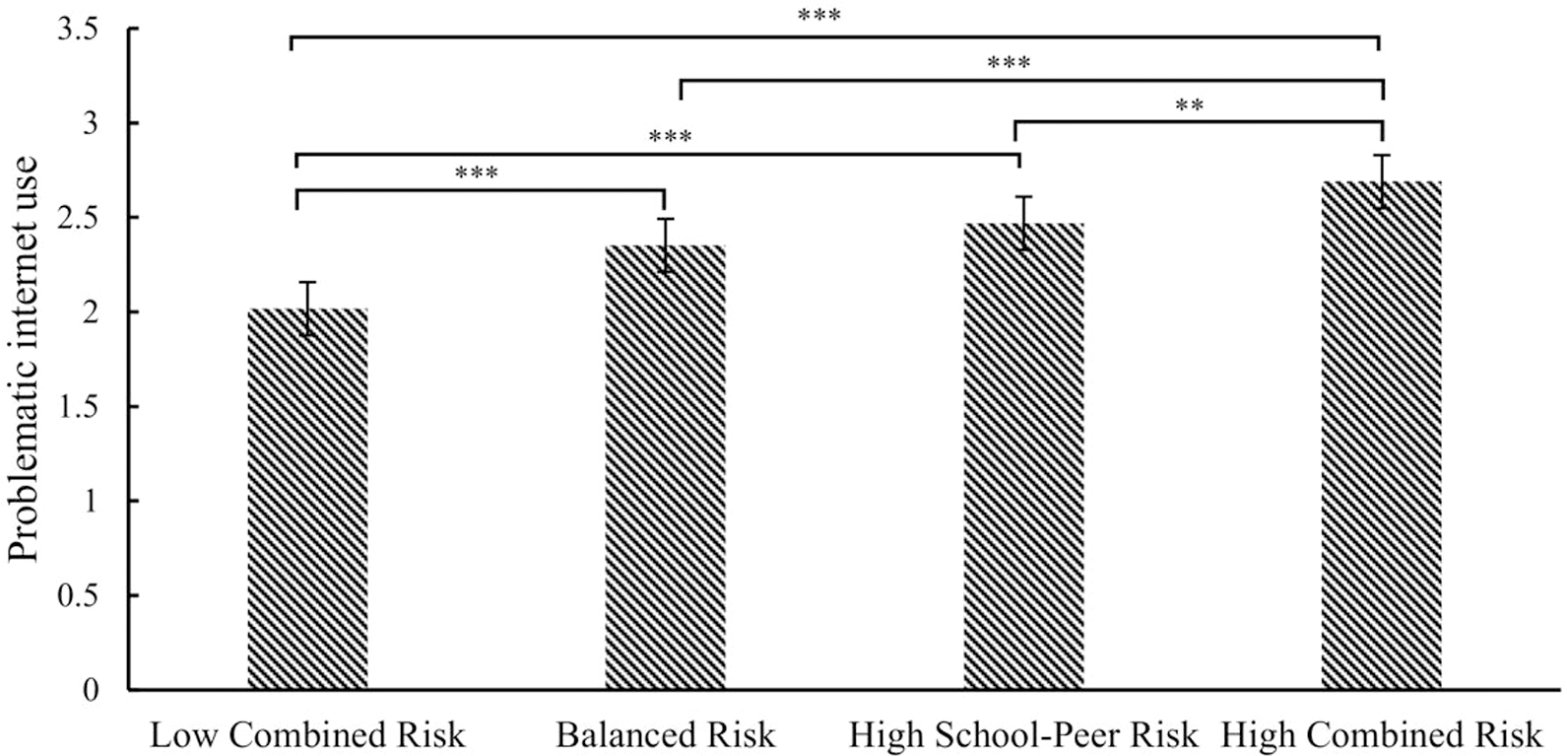
Differences in problematic Internet use scores across the joint profiles of environmental risks. Error bars represent standard errors. Brackets indicate significant Fisher’s LSD *post-hoc* pairwise comparisons. ^*^p < 0.05, ^**^p < 0.01, ^***^p < 0.001.

One-way ANOVA compared problematic Internet use differences across groups (see **Figure 3**; error bars represent standard errors). The results revealed significant differences in problematic Internet use among the four groups, *F*(3, 732) = 38.84, *p* < 0.001, η² = 0.14. Fisher’s LSD *post-hoc* comparisons showed that the High Combined Risk Group reported significantly higher problematic Internet use than the High School–Peer Risk Group, the Balanced Risk Group, and the Low Combined Risk Group. The High School–Peer Risk Group scored significantly higher than the Balanced Risk Group and the Low Combined Risk Group, and the Balanced Risk Group scored significantly higher than the Low Combined Risk Group.

### 3.5 Relative mediation: group comparisons on problematic Internet use via maladaptive cognition

Following procedures for multi-category independent variable mediation analysis (33), all continuous variables were standardized. Overall and relative mediation analyses were conducted. Using the Low Combined Risk Group as the reference, three dummy variables (D1, D2, and D3) were constructed: the Balanced Risk Group was coded 1, 0, 0; the High School–Peer Risk Group was coded 0, 1, 0; and the High Combined Risk Group was coded 0, 0, 1. After gender, age, and grade were included as covariates, the overall relative total effect was significant, *F*(3, 729) = 39.00, *p* < 0.001, and the overall relative direct effect was also significant, *F*(3, 728) = 16.76, *p* < 0.001, supporting the examination of relative mediation effects.

The relative mediation results are presented in **Table 2**. The model included gender, age, and grade as covariates. Compared with the Low Combined Risk Group, the relative indirect effect for the Balanced Risk Group was non-significant, β = 0.08, 95% CI = [−0.01, 0.16]. However, the relative indirect effects for the High School–Peer Risk Group, β = 0.23, 95% CI = [0.14, 0.33], and the High Combined Risk Group, β = 0.41, 95% CI = [0.32, 0.50], were significant. Specifically, both the High School–Peer Risk Group and the High Combined Risk Group were positively associated with problematic Internet use, and maladaptive cognition partially explained these associations.

**Table 2:** Mediation effect analysis.

Group	β	SE	LLCI	ULCI	Proportion mediated
Balanced risk group					
Relative direct effect	0.40	0.08	0.24	0.57	—
Relative mediating effect	0.08	0.04	-0.01	0.16	—
Relative total effect	0.48	0.09	0.30	0.66	—
High school–peer risk group					
Relative direct effect	0.41	0.09	0.23	0.58	—
Relative mediating effect	0.23	0.05	0.14	0.33	35.61%
Relative total effect	0.63	0.10	0.44	0.82	—
High combined risk group					
Relative direct effect	0.55	0.09	0.37	0.73	—
Relative mediating effect	0.41	0.05	0.32	0.50	42.41%
Relative total effect	0.96	0.10	0.77	1.15	—

Gender, age, and grade were included as covariates. LLCI and ULCI indicate the lower and upper limits of the 95% confidence interval, respectively. Confidence intervals for indirect effects were based on 5,000 percentile bootstrap samples. Proportion mediated was calculated as the indirect effect divided by the total effect.

### 3.6 Moderation of the direct association by psychological resilience

**Figure 4:**
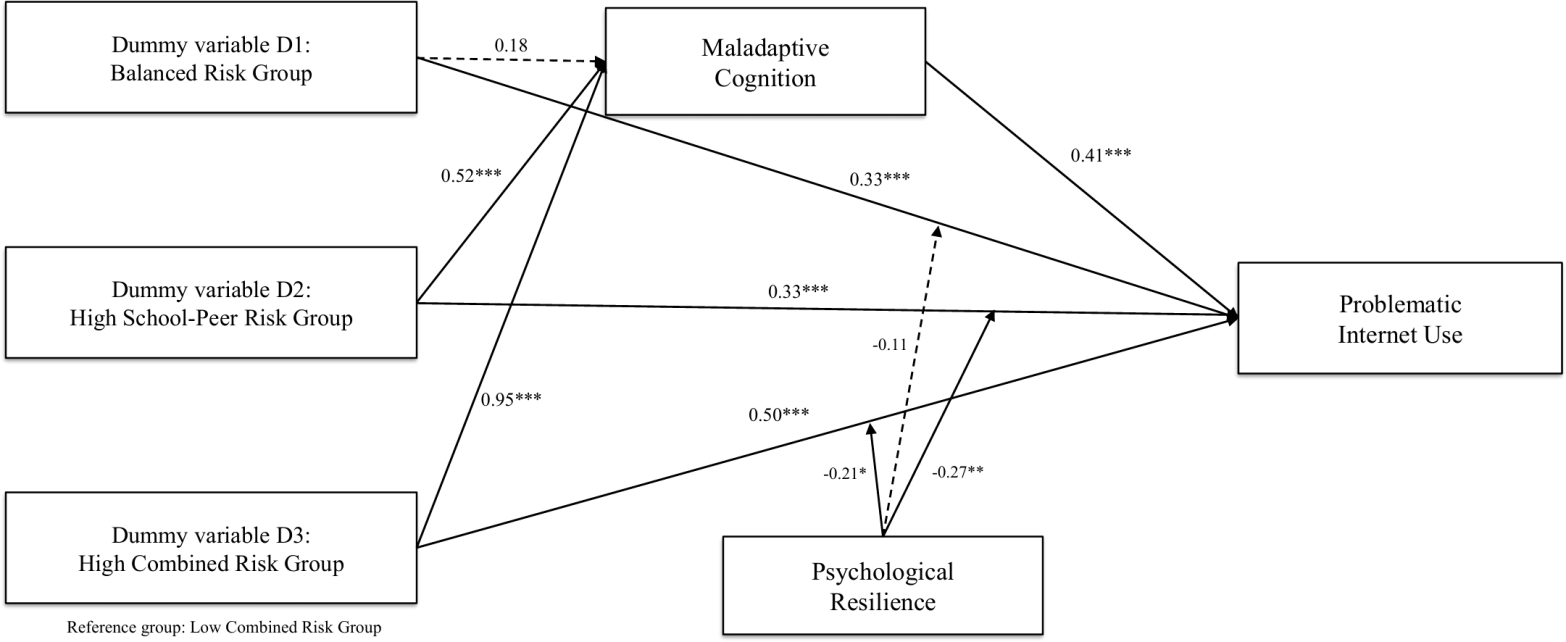
Relative mediation model with moderation. D1, D2, and D3 are dummy variables comparing the Balanced Risk Group, High School–Peer Risk Group, and High Combined Risk Group with the Low Combined Risk Group, respectively. M = maladaptive cognition; W = psychological resilience. Path coefficients are standardized coefficients. Gender, age, and grade were controlled. Solid lines indicate significant paths, and dashed lines indicate nonsignificant paths. ^*^p < 0.05, ^**^p < 0.01, ^***^p < 0.001.

As shown in **Figure 4**, PROCESS Model 5 was used to test a relative mediation model with moderation of the direct association (34). In **Figure 4**, D1, D2, and D3 represent dummy variables for the Balanced Risk Group, High School–Peer Risk Group, and High Combined Risk Group, respectively; M represents maladaptive cognition; and W represents psychological resilience. The path coefficients are standardized coefficients, and gender, age, and grade were included as covariates. Psychological resilience was examined as a moderator of the direct association between latent profile membership and problematic Internet use. The interaction terms explained additional variance in problematic Internet use, Δ*R*² = 0.009, *F*(3, 724) = 3.27, *p* < 0.05 (see **Table 3**).

**Table 3:** Moderation effect analysis.

Predictor	β	SE	*p*	LLCI	ULCI
Gender	0.03	0.06	0.660	−0.09	0.15
Age	0.10	0.05	0.037	0.01	0.19
Grade	−0.08	0.06	0.185	−0.19	0.04
D1	0.33	0.10	<0.001	0.14	0.52
D2	0.33	0.09	<0.001	0.14	0.51
D3	0.50	0.10	<0.001	0.31	0.69
*M*	0.41	0.03	<0.001	0.34	0.47
W	−0.01	0.06	0.820	−0.13	0.10
D1×W	−0.11	0.09	0.222	−0.30	0.07
D2×W	−0.27	0.09	0.003	−0.46	−0.09
D3×W	−0.21	0.10	0.035	−0.40	−0.01
*R*²	0.33				
*F*	32.40		<0.001		

D1 to D3 are dummy variables for the joint profile categories, sequentially representing the Balanced Risk Group, High School–Peer Risk Group, and High Combined Risk Group; M represents the mediator variable maladaptive cognition; W represents the moderator variable psychological resilience. Gender, age, and grade were included as covariates. The coefficients are standardized coefficients. LLCI and ULCI indicate the lower and upper limits of the 95% confidence interval, respectively.

Simple slope analyses were subsequently used to examine the association between profile membership and problematic Internet use at different levels of psychological resilience. The results indicated that when psychological resilience was low (mean − SD), the conditional direct effects were significant for the Balanced Risk Group (simple slope = 0.44, SE = 0.13, *t* = 3.52, *p* < 0.001, 95% CI = [0.19, 0.69]), the High School–Peer Risk Group (simple slope = 0.60, SE = 0.14, *t* = 4.42, *p* < 0.001, 95% CI = [0.33, 0.87]), and the High Combined Risk Group (simple slope = 0.71, SE = 0.14, *t* = 5.09, *p* < 0.001, 95% CI = [0.43, 0.98]). When psychological resilience was high (mean + SD), the conditional direct effects were non-significant for the Balanced Risk Group (simple slope = 0.21, SE = 0.14, *t* = 1.48, *p* = 0.138, 95% CI = [−0.07, 0.49]) and the High School–Peer Risk Group (simple slope = 0.05, SE = 0.13, *t* = 0.40, *p* = 0.688, 95% CI = [−0.20, 0.31]). The conditional direct effect for the High Combined Risk Group remained significant but was weaker (simple slope = 0.30, SE = 0.14, *t* = 2.15, *p* = 0.032, 95% CI = [0.03, 0.57]). These patterns suggest that psychological resilience attenuated the direct association between profile membership and problematic Internet use, particularly in the High School–Peer Risk Group. In the High Combined Risk Group, the association remained significant at high resilience, indicating that resilience may provide only limited protection when risks across multiple systems co-occur.

## 4 Discussion

Based on Ecological Systems Theory, this study used LPA to identify family–school–peer risk profiles among Chinese boarding high school students and examined how these profiles were associated with problematic Internet use. The findings showed that problematic Internet use differed across risk profiles and was highest among students in the High Combined Risk Group. Maladaptive cognition partly explained the associations between high-risk profiles and problematic Internet use, whereas psychological resilience attenuated the direct association between certain risk profiles and problematic Internet use. These findings provide a person-centered perspective for understanding heterogeneity in environmental risks among boarding students.

### 4.1 Cumulative effects of family–school–peer risks and group heterogeneity

The LPA identified four distinct risk configurations: Low Combined Risk Group (43.89%), Balanced Risk Group (20.11%), High School–Peer Risk Group (17.93%), and High Combined Risk Group (18.07%). The two high-risk profiles accounted for 36.00% of the sample, indicating that more than one-third of boarding high school students reported relatively high levels of environmental stressors. Notably, the emergence of the High School–Peer Risk Group highlights the salience of the boarding school context. Even when family risks are not uniformly elevated, pressures originating from school and peer systems may still be associated with higher problematic Internet use.

Comparisons among the latent profiles revealed a cumulative pattern in which broader exposure to environmental risks was associated with higher levels of problematic Internet use. This finding is consistent with the Demands–Resources Model (11), suggesting that adolescents facing multiple environmental demands may have fewer psychological resources available for adaptive coping. Because boarding students spend much of their daily life in school and peer contexts, school- and peer-related risks may be especially important for understanding problematic Internet use in this population. These findings suggest that prevention efforts may benefit from identifying specific risk profiles rather than focusing only on isolated risk factors.

### 4.2 Psychological processes associated with risk profiles

The present study also examined maladaptive cognition and psychological resilience as individual psychological factors related to risk-profile differences in problematic Internet use. The results showed that maladaptive cognition partially explained the associations between high-risk profiles and problematic Internet use, whereas psychological resilience attenuated the direct association between certain risk profiles and problematic Internet use. This pattern suggests that problematic Internet use among boarding students may be understood through both environmental risk configurations and individual psychological characteristics.

Specifically, maladaptive cognition served as a significant mediator for the High School–Peer Risk Group and the High Combined Risk Group. Students in these profiles reported higher maladaptive cognition, which, in turn, was associated with higher problematic Internet use. This finding is consistent with the Cognitive–Behavioral Model (12), which emphasizes the role of cognitive patterns in problematic Internet use. Boarding students exposed to higher school–peer or multi-system risks may be more likely to develop negative self-evaluations or compensatory beliefs about online activities, thereby increasing their reliance on the Internet (15).

Furthermore, psychological resilience showed a profile-specific moderating pattern. For students in the High School–Peer Risk Group, the association with problematic Internet use was significant only when resilience was low and became non-significant when resilience was high. For students in the High Combined Risk Group, the association remained significant even at high levels of resilience, although the effect was weaker. This pattern suggests that resilience may attenuate the association between school–peer risks and problematic Internet use, but its protective role may be more limited when risks from multiple systems co-occur. Therefore, individual resilience should be considered alongside broader family, school, and peer contexts rather than treated as a substitute for environmental support.

### 4.3 Potential implications for family–school–student collaboration

From an integrative perspective, the present findings may provide tentative implications for family–school–student collaboration. Because problematic Internet use was associated with heterogeneous risk profiles, prevention work may need to consider both environmental risk reduction and individual psychological support. Families and schools may work together to improve communication, strengthen school connectedness, and reduce teacher–student and peer-related stressors. At the same time, psychological support that targets maladaptive cognition and strengthens resilience may be useful for students exposed to higher environmental risks. However, because the present study did not directly test an intervention, these implications should be interpreted as preliminary directions for future prevention research rather than as evidence for a specific intervention model.

### 4.4 Limitations and future directions

Several limitations should be noted. First, cross-sectional design prevents causal conclusions. Although the findings showed significant associations among family–school–peer risk profiles, maladaptive cognition, psychological resilience, and problematic Internet use, longitudinal or experimental studies are needed to clarify temporal ordering and potential causal processes. Second, the sample was recruited from two public boarding high schools in one city in China, which may limit the generalizability of the findings. Future studies should include more diverse and representative samples from different regions and school contexts. Third, all variables were measured using self-report questionnaires, which may introduce common method bias and social desirability effects. Although Harman’s single-factor test suggested that common method bias was unlikely to seriously distort the present findings, future research would benefit from multi-informant data from parents, teachers, or peers. Fourth, although established instruments or measures previously used in adolescent samples were adopted in the present study, the factor structure and measurement stability of these tools among boarding high school students require further examination. Future studies should further evaluate the construct validity, measurement invariance, and latent structural relations among these variables in more diverse boarding student samples, preferably using longitudinal or structural equation modeling approaches. Finally, some potentially relevant contextual variables, such as school management style, dormitory climate, parental migration status, and Internet access conditions, were not fully assessed. Future research should incorporate these contextual factors to better understand problematic Internet use among boarding students.

## 5 Conclusion

The present study identified four latent profiles of family–school–peer risks among boarding high school students: Low Combined Risk, Balanced Risk, High School–Peer Risk, and High Combined Risk. Problematic Internet use differed across these profiles and was highest in the High Combined Risk Group. Maladaptive cognition partially explained the associations between high-risk profiles and problematic Internet use. Psychological resilience attenuated the direct association between certain risk profiles and problematic Internet use, particularly in the High School–Peer Risk Group. These findings suggest that problematic Internet use among boarding students is associated with heterogeneous configurations of environmental risks and individual psychological characteristics.

## Data Availability

The raw data supporting the conclusions of this article will be made available by the authors, without undue reservation.
